# Efficacy and safety of pegylated recombinant human granulocyte colony-stimulating factor as a support for chemotherapy for gestational trophoblastic neoplasia: a propensity score matching analysis

**DOI:** 10.3389/fonc.2025.1590189

**Published:** 2025-09-22

**Authors:** Xin Zhao, Hongwei Zhao, Lixin Sun, Lijuan Yan, Haiqiong Han, Zhiguo Li

**Affiliations:** ^1^ Department of Gynecologic Tumor, Shanxi Province Cancer Hospital, Shanxi Hospital Affiliated to Cancer Hospital, Chinese Academy of Medical Sciences, Cancer Hospital Affiliated to Shanxi Medical University, Taiyuan, Shanxi, China; ^2^ Department of Hepatopancreatobiliary Surgery, Shanxi Province Cancer Hospital, Shanxi Hospital Affiliated to Cancer Hospital, Chinese Academy of Medical Sciences, Cancer Hospital Affiliated to Shanxi Medical University, Taiyuan, Shanxi, China

**Keywords:** gestational trophoblastic neoplasia, chemotherapy, hematological toxicity, febrile neutropenia, efficacy

## Abstract

**Background:**

Gestational trophoblastic neoplasia (GTN) is a highly malignant tumor that can be effectively treated with chemotherapy alone. Recombinant human granulocyte colony-stimulating factor (rhG-CSF) is used to reverse the adverse effects of chemotherapy, but its half-life is short, requiring daily injections and significantly reducing patient tolerance rates. Pegylated recombinant human granulocyte colony-stimulating factor (PEG-rhG-CSF) can be administered for a longer duration, improving patient tolerance and compliance. This retrospective study was designed to investigate the efficacy and safety of PEG-rhG-CSF for preventing hematological toxicity after chemotherapy for GTN.

**Methods:**

We retrospectively assessed 200 GTN patients treated with chemotherapy from January 2019 to December 2021. One hundred patients received 6 mg of PEG-rhG-CSF within 24 h after chemotherapy and composed the experimental group. One hundred additional patients who were treated with recombinant human granulocyte colony-stimulating factor were matched 1:1 via the propensity score matching method and served as the control group. The main observations were differences in hematological toxicity, neutrophil changes, febrile neutropenia incidence and adverse reactions.

**Results:**

The incidences of grade 3/4 neutropenia, grade 4 neutropenia, febrile neutropenia, antibiotic use, chemotherapy delay and bone pain in the experimental group were significantly lower than those in the control group (*P <* 0.05). The duration of grade 3/4 neutropenia in the experimental group was significantly shorter than that in the control group (3.6 days vs. 6.5 days, *P* < 0.05). The incidence rates of adverse events in the experimental group and control group were 51% and 77%, respectively, and the difference was statistically significant (*P* = 0.006).

**Conclusion:**

PEG-rhG-CSF has good efficacy and safety in preventing hematological toxicity in GTN patients after chemotherapy.

## Introduction

Gestational trophoblastic neoplasia (GTN) refers to a group of malignant tumors formed by the abnormal proliferation of trophoblastic cells that can occur after a mole pregnancy or a nonmole pregnancy (1). GTNs include the following histological types: invasive mole (IM), choriocarcinoma (CC), placental site trophoblastic tumor, and epithelioid trophoblastic tumor (2). A study by Epstein and Joneborg (3) revealed that GTN occurred in women of childbearing age, and its incidence was closely related to pregnancy; 50% of patients occurred from mole and 25% from abortion. Chemotherapy is a common treatment for GTN patients (1). With the help of chemical drugs, malignant tumor cells can be killed to prolong the life of patients, and most patients can be cured by chemotherapy (4). According to the 2018 International Federation of Gynecology and Obstetrics (FICO) Cancer Report, patients with scores of 0 to 6 can receive single-agent chemotherapy, and commonly used drugs include methotrexate (MTX), actinomycin D, and 5-fluorouracil, whereas patients with scores of ≥ 7 and a pathological diagnosis of choriocarcinoma are recommended for combination chemotherapy (5). The main side effects of chemotherapy include bone marrow suppression, nausea and vomiting, liver and kidney function injury and mucosal injury, and rare side effects include cardiopulmonary toxicity and neurotoxicity (6). During chemotherapy, neutropenia is the most common hematological toxicity and easily induces febrile neutropenia (FN), toxic shock from infection, and even death, which seriously affects the clinical treatment and survival of patients with tumors and increases medical costs (7–9).

Recombinant human granulocyte colony-stimulating factor (rhG-CSF) is an effective drug for preventing and treating bone marrow suppression caused by tumor chemotherapy, but conventional rhG-CSF is a short-acting drug that requires daily injection in general patients (10, 11). The human granulocyte stimulating factor produced by the use of polyethylene glycol and gene recombination technology has a long-term effect on reducing plasma clearance and prolonging the half-life of the fungus (12). Studies have shown that Pegylated recombinant human granulocyte colony-stimulating factor (PEG-rhG-CSF) can reduce the occurrence of neutropenia and improve the safety of chemotherapy in patients receiving multiple cycles of chemotherapy (13, 14). Compared with rhG-CSF, PEG-rhG-CSF significantly reduces the number of repeat injections and thus has the advantages of fewer injections and fewer side effects. It can prevent the occurrence of FN, improve the quality of life of chemotherapy patients, and reduce the risk of chemotherapy delay (15, 16). However, the clinical application of PEG-rhG-CSF in GTN chemotherapy still lacks data. To explore whether PEG-rhG-CSF could be applied to the GTN after chemotherapy, a retrospective, cohort-controlled trial was conducted to assess the efficacy and safety of PEG-rhG-CSF for preventing hematological toxicity in the GTN after chemotherapy.

## Methods

### Patients

This was a retrospective cohort study. This study was approved by the Ethics Committee of Shanxi Province Cancer Hospital. All participants signed a written informed consent form, and all methods were performed in accordance with the Declaration of Helsinki.

Clinical data were collected retrospectively from January 2019 to December 2021 from patients diagnosed with GTN and treated with chemotherapy at Shanxi Cancer Hospital. One hundred patients met the inclusion criteria and received PEG-rhG-CSF within 24 h after the end of each cycle of chemotherapy. This was defined as prophylactic use, and these patients composed the experimental group. We used a propensity score matching method (PSM) to match 100 (1:1) out of 380 patients by various control factors, including age, body mass index (BMI), Eastern Cooperative Oncology Group (ECOG) performance status, history of smoking, history of alcohol consumption, hypertension, diabetes, heart disease, absolute neutrophil count (ANC) baseline and pathological stage and score. The control group was given a subcutaneous injection of rhG-CSF (5 µg/kg) once a day for 10 days at 24 h after chemotherapy. If the absolute neutrophil count (ANC) was > 1.5×10^9^/L, the injection was stopped.

The inclusion criteria were as follows: 1. had a GTN confirmed by pathological biopsy. 2. an estimated survival time > 3 months. 3. a white blood cell count > 4×10^9^/L and ANC > 2×10^9^/L before chemotherapy. 4. were aged 18–50 years and had a Karnofsky Performance Scale > 80 points. 5. had a FIGO 2000 Gross Cancer Network clinical staging and prognosis score ≥4 points. 6. without liver or kidney dysfunction or other malignant tumours. Patients were excluded from the study if they met the following criteria: 1. met the inclusion of radiation therapy 4 weeks prior to enrollment (excluding local radiation therapy for bone metastases). 2. had a hematopoietic stem cell transplant or a bone marrow transplant. 3. had chemotherapy received within 4 weeks before enrollment. 4. were diagnosed with bone marrow metastasis. 5. had severe, uncontrolled diabetes. 6. had participated in other clinical trials at the same time. 7. had an active infection. 8. had a serious heart, kidney, liver or other vital organ chronic disease.

### Chemotherapy regimen

The treatment of GTN is based on the FIGO prognostic scoring system, in which those with a score of ≤ 6 are low-risk GTN and single-drug chemotherapy is adopted; those with a score of ≥ 7 are high-risk GTN and combination chemotherapy is adopted. The regimens of single drug and combination chemotherapy in this study are as follows.

MTX monotherapy regimen: MTX 0.4 mg/kg daily (maximum dose 25 mg/day), intravenous or intramuscular injection, once daily for days 1-5, repeated every 14 days. EMA-CO combination chemotherapy regimen: actinomycetin-D + etoposide + methotrexate (EMA) + vincristine + cyclophosphamide (CO), EMA on day l and day 2, CO on day 8 and day 15 for the next course of treatment.

### Dosing regimen

In the experimental group, 6 mg of PEG-rhG-CSF was injected subcutaneously (Qilu Pharmaceutical Co., Ltd.) within 24 h after the end of each cycle of chemotherapy. The control group was given a subcutaneous injection of rhG-CSF (5 µg/kg; Qilu Pharmaceutical Co., Ltd.) once a day for 10 days at 24 h after chemotherapy. If the ANC > 1.5×10^9^/L was checked two consecutive times, the injection was stopped.

### Study outcomes

The primary outcome was the incidence of grade 3/4 neutropenia (defined as ANC < 0.5×10^9^/L). The secondary outcomes were the duration of grade 3/4 neutropenia (according to the National Cancer Institute common toxicity criteria, V4.03), incidence of FN, antibiotic utilization rate, chemotherapy delay, incidence of bone pain and adverse reactions. Data on the aforementioned parameters were collected during one cycle of first-line chemotherapy.

### Statistical analysis

All the statistical analyses were performed with SPSS software version 26.0 for Windows (SPSS, Chicago, IL, USA). Normally distributed data were analysed by Student’s t test, and the data are expressed as the means ± standard deviations. If the data were not normally distributed, the nonparametric rank-sum test was applied to analyse the measured data. The chi-square test was applied to the categorical data. *P* < 0.05 was considered to indicate statistical significance.

## Results

### Basic information

**Figure 1:**
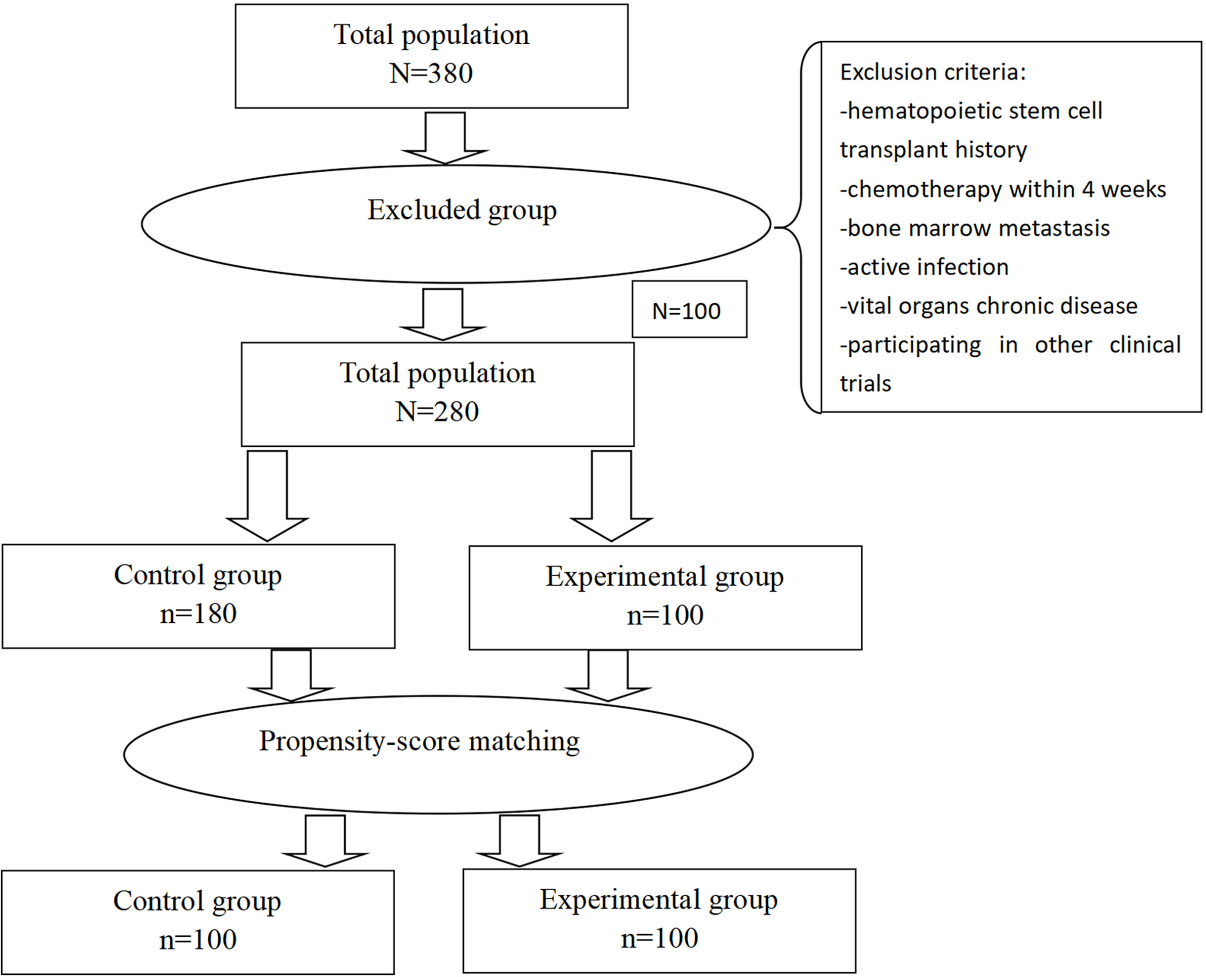
Plot of patient selection and propensity score matching. The 100 patients who received recombinant human granulocyte colony-stimulating factor (rhG-CSF) were matched to 100 patients who received Pegylated recombinant human granulocyte colony-stimulating factor (PEG-rhG-CSF) in terms of age, body mass index, ECOG performance status, smoking history, alcohol consumption, hypertension, diabetes, heart disease, absolute neutrophil count (ANC) baseline and pathological stage and score.

In total, 380 patients were enrolled in this study, and 100 were excluded on the basis of the following exclusion criteria: 15 patients who received chemotherapy within 4 weeks before enrollment, 20 patients who had severe heart and kidney disease, 30 patients who lacked clinical data, and 35 patients who were lost to follow-up. The remaining 280 patients were included in our study. The baseline characteristics of experimental group versus control group before and after PSM analysis are summarised in **Table 1**. Before the PSM analysis, all the patients were analysed, and several significant differences between the two groups were seen. After PSM, two well-balanced groups of 100 patients were analysed (**Table 1** and **Figure 1**). There was no significant difference in the baseline demographic characteristics between the experimental and control groups.

**Table 1:** Baseline characteristics of the patients in experimental group and control group according to propensity score matching (PSM).

Characteristic	Before PSM			After PSM		
	Experimental group (n=100)	Control group (n=180)	*P*	Experimental group (n=100)	Control group (n=100)	*P*
Age (years)	39.27 ± 4.46	39.81 ± 5.12	0.031	39.27 ± 4.46	39.25 ± 4.34	0.921
BMI (kg/m^2^)	24.46 ± 2.68	24.71 ± 2.60	0.893	24.46 ± 2.68	24.51 ± 2.48	0.549
ECOG performance status			0.490			0.882
0	66	126		66	65	
1	34	54		34	35	
Smoking history			0.655			0.651
Smoker	12	25		12	10	
Never smoker	88	155		88	90	
Alcohol consumption			0.036			0.637
Yes	9	33		9	11	
No	91	147		91	89	
Hypertension			0.285			0.777
Yes	55	87		55	53	
No	45	93		45	47	
Diabetes			0.488			0.653
Yes	32	65		32	35	
No	68	115		68	65	
Heart disease			0.458			0.718
Yes	20	30		20	18	
No	80	150		80	82	
ANC baseline (×10^9^/L)	4.43 ± 0.67	4.34 ± 0.55	0.041	4.43 ± 0.67	4.31 ± 0.62	0.393
Histopathological type			0.633			0.767
Invasive mole	64	110		64	66	
Choriocarcinoma	36	70		36	34	
FIGO staging			0.907			0.751
I	10	18		10	8	
II	31	50		31	34	
III	54	100		54	50	
IV	5	12		5	8	
Prognostic score			0.046			0.617
0-6	22	60		22	25	
≥7	78	120		78	75	

### FIGO staging and prognosis score

Among the 200 GTN patients included, 130 had IM, 70 had CC, and the number of IM was approximately twice that of CC. According to FIGO staging and prognostic scoring criteria (2000), among the 200 GTN patients, 18 had stage I, 65 had stage II, 104 had stage III, and 13 had stage IV disease. There were 47 low-risk patients with scores ranging from 0–6 and 153 high-risk patients with scores of ≥7. There was no significant difference in stage or score indicators between the two groups of patients. The stages and grades are shown in **Table 1**.

### The incidence and duration of grade 3–4 neutropenia

**Figure 2:**
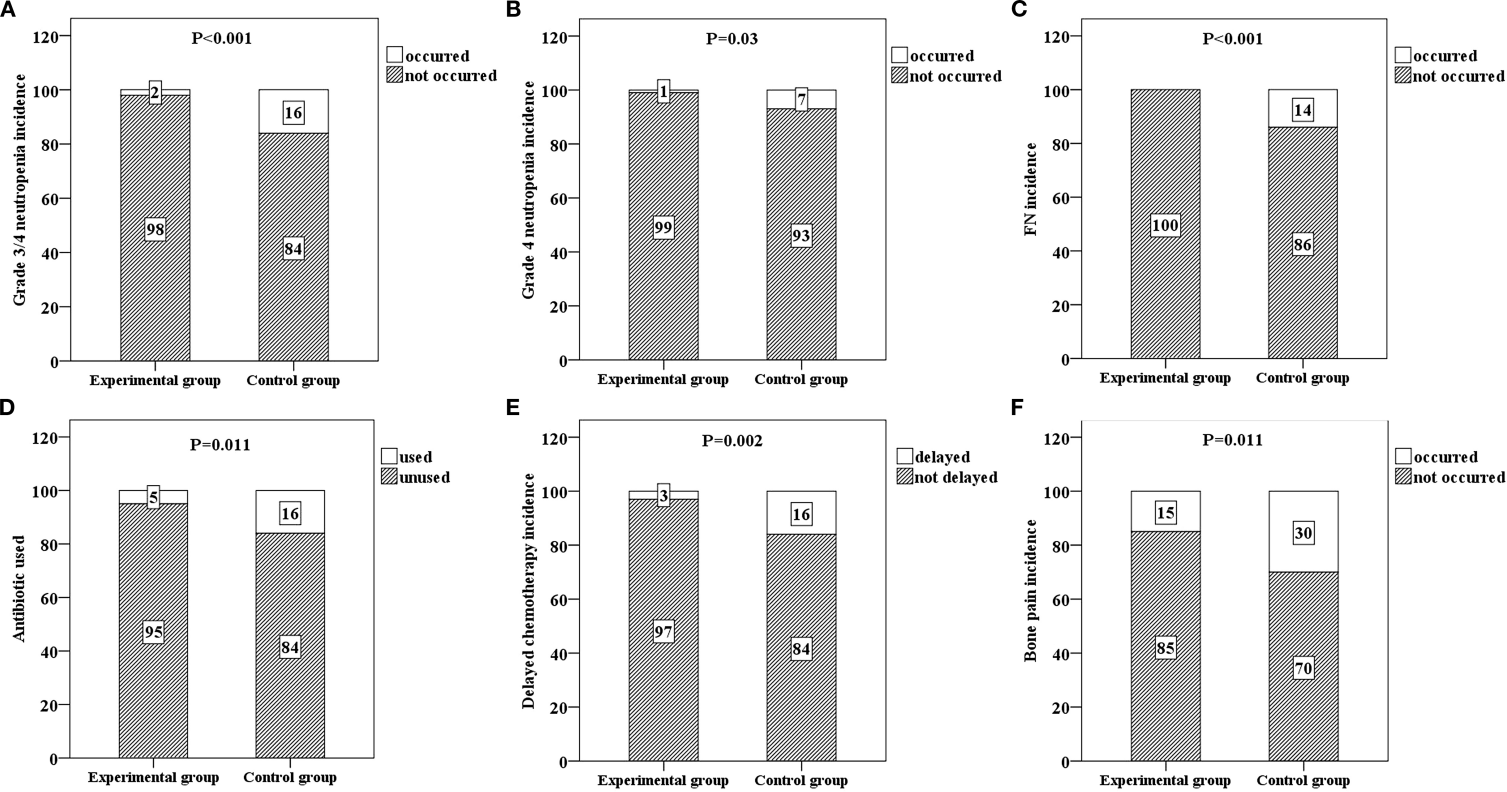
Six bar charts respectively show the incidence rates and comparison results of various events in the experimental group and the control group. **(A)** shows the incidence rate of grade 3/4 neutropenia in both groups (*p* < 0.001), **(B)** shows the incidence rate of grade 4 neutropenia in both groups (*p* = 0.03), **(C)** shows the incidence rate of febrile neutropenia (FN) in both groups (*p* < 0.001), **(D)** shows the antibiotic usage situation in both groups (*p* = 0.011), **(E)** shows the incidence rate of chemotherapy delay in both groups (*p* = 0.002), and **(F)** shows the incidence rate of bone pain in both groups (*p* = 0.011). Each situation is labeled with numbers within the bar charts.

The primary endpoint, grade 3/4 neutropenia, was significantly lower in the experimental group (2/100, 2%) than in the control group (16/100, 16%) (*P* < 0.001) (**Table 2** and **Figure 2A**). The average duration of grade 3/4 neutropenia in the experimental group was 3.55 days, whereas that in the control group was 6.32 days (**Table 2**). The duration of grade 3/4 neutropenia in the experimental group was significantly shorter than that in the control group, and the difference between the two groups was also statistically significant (*P* = 0.04) (**Table 2**).

**Table 2:** Analysis of endpoints.

Endpoints	Experimental group (n=100)	Control group (n=100)	*P*
Grade 3/4 neutropenia occurs			<0.001
Yes	2	16	
No	98	84	
The duration of grade 3/4 neutropenia			0.04
n	2	16	
Median (Q1, Q3)	3.6(3.5,3.6)	6.5(4.9,7.5)	
Grade 4 neutropenia occurs			0.03
Yes	1	7	
No	99	93	
FN occurs			<0.001
Yes	0	14	
No	100	86	
Antibiotic used			0.011
Yes	5	16	
No	95	84	
Chemotherapy delayed			0.002
Yes	3	16	
No	97	84	
Bone pain occurs			0.011
Yes	15	30	
No	85	70	

### The incidence of grade 4 neutropenia and the incidence of FN

The incidence rates of grade 4 neutropenia in the experimental and control groups were 1% and 7%, respectively, and the difference between the two groups was statistically significant (*P* = 0.03) (**Table 2** and **Figure 2B**). There were no FN cases in the experimental group (0%) or 14 FN cases in the control group (14%), and the difference between the two groups was statistically significant (*P* < 0.001) (**Table 2** and **Figure 2C**).

### Influences on chemotherapy delay, antibiotic use and the occurrence of bone pain

Patients in the experimental group had no dose reduction of chemotherapy drugs, and only three patients experienced treatment delays after being given PEG-rhG-CSF. This could be completely improved after secondary prevention with PEG-rhG-CSF. The rate of antibiotic use in the experimental group was significantly lower than that in the control group (*P* = 0.011) (**Table 2** and **Figure 2D**). A total of 3 patients in the experimental group had delayed chemotherapy due to granulocytopenia and vomiting, 16 patients in the control group had delayed chemotherapy, and the difference between the two groups was statistically significant (*P* = 0.002) (**Table 2** and **Figure 2E**). The incidence of bone pain in the experimental group (15%) was significantly lower than that in the control group (30%) (*P* = 0.011) (**Table 2** and **Figure 2F**).

### Adverse events

The total incidence rates of additional adverse reactions in the experimental group and the control group were 51% and 70%, respectively, indicating that PEG-rhG-CSF reduced side effects (*P* = 0.006). Among the adverse reactions reported in both groups, the most common adverse events included vomiting, oral mucositis, diarrhea, constipation, and anorexia (**Table 3**).

**Table 3:** Analysis of adverse reactions.

Adverse events	Experimental group (n=100)	Control group (n=100)
At least one adverse reaction occurred	51	70
χ2	7.55	
*P*	0.006	
Vomiting	28	35
Oral mucositis	15	20
Diarrhea	10	8
Fever	0	14
Anemia	5	8
Hypokalemia	5	9
Myocardial ischemia	0	0
Dizziness	2	3
Thrombocytopenia	5	6
Palpitation	1	3
Fatigue	5	8
Constipation	8	10
Abdominal distention	5	6
Anorexia	6	10

## Discussion

The GTN is highly capable of erosion, destruction of blood vessels, and early blood metastasis and can involve tissues and organs throughout the body. Therefore, GTN can be treated with systemic chemotherapy, and solid tumors can be cured by chemotherapy. Low-risk patients are treated with a single chemical agent, methotrexate or actinomycin D, which results in a remission rate close to 100% (4). The treatment remission rates for high-risk patients are 80-90%, and those for ultrahigh-risk patients are 68% (5, 17). However, chemotherapy-related bone marrow suppression limits the implementation of chemotherapy. In addition, chemotherapy-induced agranulocytosis, infection and other factors are the main reasons for delayed chemotherapy and increased expenditure (18, 19).

rhG-CSF is an effective drug for preventing and treating bone marrow suppression caused by tumor chemotherapy (10, 20, 21). rhG-CSF promotes the proliferation and differentiation of granulocyte progenitor cells and enhances the functional activity of mature neutrophils, thereby reducing the incidence of FN in patients undergoing chemotherapy. However, due to its relatively low molecular weight, rhG-CSF is primarily eliminated via renal excretion and exhibits a short elimination half-life of only 3.5 hours (22). Therefore, rhG-CSF is a short-acting drug that requires daily injection in general patients (11, 23, 24). PEG-rhG-CSF is synthesized by selectively conjugating a 20 kDa polyethylene glycol (PEG) molecule to the N-terminal end of the rhG-CSF protein. As a result of the increased molecular weight, PEG-rhG-CSF is less readily cleared by the kidneys, and its primary elimination pathway shifts to neutrophil-mediated clearance. This modification significantly extends the plasma half-life to approximately 47 hours, thereby allowing for a single dose per chemotherapy cycle to effectively prevent neutropenia (22). PEG-rhG-CSF can reduce the occurrence of neutropenia and improve the safety of chemotherapy in patients receiving multiple cycles of chemotherapy (12, 23). Compared with rhG-CSF, PEG-rhG-CSF significantly reduces the number of repeat injections and thus has the advantages of fewer injections and fewer side effects. It can prevent the occurrence of FN, improve the quality of life of chemotherapy patients, and reduce the risk of chemotherapy delay (23, 24). A single-centre retrospective study reported that pegfilgrastim (a long-acting G-CSF) was useful for maintaining a relative dose intensity within the required therapeutic range for the treatment of endometrial cancer and reducing the number of hospital visits (25). In addition, in a single-institution retrospective review of 46 patients with ovarian or primary peritoneal cancer who received prophylactic pegfilgrastim on the same day, no patients experienced FN episodes, hospitalizations or antibiotic use secondary to neutropenia nor did they experience dose reductions or chemotherapy delays due to neutropenia (26). Furthermore, a phase III randomized controlled study revealed that the use of long-acting G-CSF resulted in a reduction in the number of clinical visits for newly diagnosed patients with epithelial ovarian cancer (27). However, the clinical application of PEG-rhG-CSF in GTN chemotherapy still lacks data. This study provides data supporting the clinical use of PEG-rhG-CSF in GTN chemotherapy.

Clinical studies (28) have shown that a single injection of PEG-rhG-CSF can increase the absolute value of neutrophils, the efficacy of PEG-rhG-CSF is better than that of traditional rhG-CSF, and the drug effect lasts longer. In this study, the incidence of grade 3/4 neutropenia was lower in the experimental group than in the control group. The median duration of grade 3/4 neutropenia was only 3.5 days in the experimental group, whereas it was only 6.5 days in the control group. In addition, grade 4 neutropenia occurred in only one patient in the experimental group, whereas seven patients in the control group did. PEG-rhG-CSF acts on hematopoietic cells by binding to the surface receptors of these cells. It promotes the differentiation and proliferation of granulocyte progenitor cells into neutrophils, induces their maturation, and enhances the survival and function of mature neutrophils. As a result, it increases the dose-dependent level of neutrophils. The pharmacokinetic model shows that the ANC curve after subcutaneous administration of PEG-rhG-CSF presents a bimodal shape. PEG-rhG-CSF can stimulate the release of relatively mature neutrophils into the peripheral blood, causing an increase in ANC and resulting in the first peak, which generally takes effect 12 to 24 hours after administration. Subsequently, the bone marrow suppression caused by chemotherapy leads to a decrease in ANC, Meanwhile, the blood - drug concentration of PEG-rhG-CSF can remain until hematopoietic function begins to recover, promoting the proliferation and differentiation of primitive granulocytes into new neutrophils, causing an increase in ANC and the appearance of the second peak. When ANC returns to the normal value, the clearance rate of PEG-rhG-CSF increases, and it is quickly eliminated from the body (29).

These findings suggest that the prophylactic use of PEG-rhG-CSF can sustainably stabilize neutrophil levels. Neutropenia leads to a significant increase in infection. In previous studies, after a week of ANC decline, the probability of infection caused by grade 3/4 neutropenia ranged from 10% to 30% (30, 31). In the present study, the experimental group received fewer antibiotics than the control group did, indicating a lower incidence of infection, which is closely related to a lower incidence of grade 3/4 neutropenia. None of the patients in the experimental group developed FN, whereas 14 patients in the control group did. In actual clinical applications, the duration of rhG-CSF usage is often shorter. An observational study conducted in community hospitals in the United States revealed that chemotherapy patients received an average of 3.7 days and 4.6 days of rhG-CSF prevention during the first chemotherapy cycle and subsequent cycles, respectively. All patients using PEG-rhG-CSF completed a full single - dose per cycle. Compared with using conventional rhG-CSF prophylaxis, the absolute risk of FN increased by 1.8% and the relative risk increased by 41% (*P* = 0.040) (32).

Our data showed that prophylactic use of PEG-rhG-CSF is effective at preventing delays in chemotherapy cycles. The application of PEG-rhG-CSF can effectively reduce the incidence of infection by preventing neutropenia while also reducing the incidence of FN and delaying chemotherapy (15, 16). These findings are also consistent with a meta-analysis of 20 clinical studies that showed that primary prevention with PEG-rhG-CSF significantly reduced the risk of FN (33).

According to the recommendation of the National Comprehensive Cancer Network guidelines, a dose of 6 mg is suggested for the clinical use of PEG-rhG-CSF (34, 35). We used 6 mg of PEG-rhG-CSF according to the established guidelines. In the present study, compared with that in the control group, the incidence of bone pain was 15%, and it remained constant at 30%. Our data revealed that the main adverse effect of PEG-rhG-CSF was bone pain, which is consistent with the results of a previous study (35). The main cause of bone pain is the inflammatory reaction that occurs during the quantitative and qualitative expansion of the bone marrow. For mild to moderate bone pain, no treatment is necessary (36). For severe pain, acetaminophen and non-steroidal anti-inflammatory drugs can be used. Antihistamines or opioid drugs can also be used for symptomatic treatment, or the dosage of PEG-rhG-CSF can be reduced (37). However, compared with rhG-CSF, PEG-rhG-CSF significantly reduced the incidence of bone pain. In addition, adverse effects such as vomiting, oral mucositis, diarrhea, fever, anemia, hypokalaemia, myocardial ischemia, dizziness, thrombocytopenia, palpitation, fatigue, constipation, abdominal distention and anorexia are considered to be caused by chemotherapy. These findings suggested that the prophylactic use of PEG-rhG-CSF significantly reduced the incidence of adverse chemical reactions.

## Limitations

This retrospective cohort study has two limitations. First, retrospective studies have a lower level of evidence than prospective studies. Second, this was a single-center study with a small sample size, which could be improved by having a large sample size and multiple centers. However, to the best of our knowledge, this is the first retrospective study of PEG-rhG-CSF as a support for GTN chemotherapy. These encouraging data led us to plan a prospective randomized clinical trial of PEG-rhG-CSF for the treatment of chemotherapy-induced neutropenia and FN in GTN patients.

## Conclusion

Overall, our study demonstrated that PEG-rhG-CSF can be used as a prophylactic treatment for chemotherapy-induced neutropenia. PEG-rhG-CSF is known for its excellent safety, low incidence of adverse reactions and simple application. A single dose of PEG-rhG-CSF was found to be effective at reducing the occurrence of grade 3/4 neutropenia, FN, and delayed chemotherapy in patients receiving GTN chemotherapy. This provides a new option for controlling neutropenia during chemotherapy in GTN patients.

## Data Availability

The original contributions presented in the study are included in the article/Supplementary Material. Further inquiries can be directed to the corresponding author.
